# An ES-Like Pluripotent State in FGF-Dependent Murine iPS cells

**DOI:** 10.1371/journal.pone.0016092

**Published:** 2010-12-30

**Authors:** Bruno Di Stefano, Christa Buecker, Federica Ungaro, Alessandro Prigione, Hsu-Hsin Chen, Maaike Welling, Maureen Eijpe, Gustavo Mostoslavsky, Paul Tesar, James Adjaye, Niels Geijsen, Vania Broccoli

**Affiliations:** 1 Stem Cells and Neurogenesis Unit, Division of Neuroscience, San Raffaele Scientific Institute, Milan, Italy; 2 Center for Regenerative Medicine, Harvard Medical School, Massachusetts General Hospital, Boston, Massachusetts, United States of America; 3 Harvard Stem Cell Institute, Harvard University, Cambridge, Massachusetts, United States of America; 4 Molecular Embryology and Aging Group, Department of Vertebrate Genomics, Max Planck Institute for Molecular Genetics, Berlin, Germany; 5 Department of Cell Biology, Erasmus Medical Centre, Rotterdam, The Netherlands; 6 Department of Stem Cell Biology and Regenerative Medicine, Boston University School of Medicine, Boston, Massachusetts, United States of America; 7 Department of Genetics, Case Western Reserve University, Cleveland, Ohio, United States of America; 8 Hubrecht Institute for Developmental Biology and Stem Cell Research, Utrecht, The Netherlands; 9 Fondazione IRCCS Policlinico San Matteo, Pavia, Italy; 10 Department of Clinical Sciences of Companion Animals, Utrecht University Veterinary School, Utrecht, The Netherlands; University of Southern California, United States of America

## Abstract

Recent data demonstrates that stem cells can exist in two morphologically, molecularly and functionally distinct pluripotent states; a naïve LIF-dependent pluripotent state which is represented by murine embryonic stem cells (mESCs) and an FGF-dependent primed pluripotent state represented by murine and rat epiblast stem cells (EpiSCs). We find that derivation of induced pluripotent stem cells (iPSCs) under EpiSC culture conditions yields FGF-dependent iPSCs from hereon called FGF-iPSCs) which, unexpectedly, display naïve ES-like/ICM properties. FGF-iPSCs display X-chromosome activation, multi-lineage differentiation, teratoma competence and chimera contribution *in vivo*. Our findings suggest that in 129 and Bl6 mouse strains, iPSCs can dominantly adopt a naive pluripotent state regardless of culture growth factor conditions.

Characterization of the key molecular signalling pathways revealed FGF-iPSCs to depend on the Activin/Nodal and FGF pathways, while signalling through the JAK-STAT pathway is not required for FGF-iPS cell maintenance. Our findings suggest that in 129 and Bl6 mouse strains, iPSCs can dominantly adopt a naive pluripotent state regardless of culture growth factor conditions.

## Introduction

Pluripotent stem cells are characterized by their ability to expand indefinitely *in vitro* while retaining the capacity to generate derivatives of all three germ layers, both *in vitro* and *in vivo*. Sources of pluripotent stem cells include blastocyst embryos, which give rise to embryonic stem cells (ES cells), and the post-implantation epiblast which gives rise to epiblast stem cells (EpiSCs) [1], [2].

ES cells and EpiSCs are both pluripotent as they are capable of generating derivatives of the three embryonic germ layers upon *in vitro* or *in vivo* differentiation, yet important molecular and functional differences exist between these two pluripotent states. At the molecular level, the ES cell pluripotent state is maintained by a combination of LIF/JAK/STAT3 and BMP4 signaling, while EpiSCs require a combination of bFGF and TGFβ/Activin signaling for their continued self-renewal. The different culture conditions that maintain ES cells and EpiSCs are reflected in the morphological, molecular and functional properties of these cells. Murine ES cells form dome-shaped three dimensional colonies and are capable of generating chimeras with functional contribution to all somatic lineages as well as the germline. In contrast, EpiSCs form flatted colonies that are split by mechanical- or collagen-mediated passaging as small clusters of cells, since EpiSCs cannot be passaged as single cells by trypsin digest. EpiSCs are pluripotent and form derivatives of all three germ layers during in vitro differentiation and upon teratoma formation in vivo. Unlike ES cells, EpiSCs can even generate trophoectoderm derivatives *in vitro*. Yet, fail to integrate with the ICM upon morula aggragation and as a result, chimera forming potential of EpiSCs is very low or even absent. Thus, while EpiSCs are pluripotent, to-date their *in vivo* developmental potential is limited to teratoma formation.

Above results demonstrate that in the mouse, two functionally distinct pluripotent states exist, a naïve LIF-dependent pluripotent state that is compatible with the pre-implantation ICM and a primed FGF-dependent state that is reminiscent of the post-implantation epiblast [3].

The ability to generate ES cell lines is restricted to only a few inbred mouse strains whereas other, so-called “non-permissive” mouse strains fail to yield ES cells under standard culture conditions, but instead can give rise to to EpiSCs,Pluripotent stem cell lines from other species, including human and rat, share many of the defining characteristics of EpiSCs, suggesting that the EpiSC pluripotent state is the common stable pluripotent state for most strains of mice as well as other species. Interestingly, Hanna and colleagues recently demonstrated that the constitutive ectopic expression of either Klf4 or cMyc allows the derivation of LIF-dependent ES-like cells from blastocyst embryos of the non-permissive NOD mouse strain [4]. In addition, LIF/serum-dependent ES-like cell lines can be generated through somatic cell reprogramming of NOD fibroblasts with defined factors (Oct4, Sox2, Klf4, cMyc) that have recently been shown to allow the generation of induced pluripotent stem cells (iPS cells) from somatic cells [5], [6]. Yet, as with the blastocyst-derived NOD ES cell lines, the stable propagation of NOD iPS cells is dependent on the continued ectopic expression of Klf4 or cMyc.

Small molecule inhibitors of glycogen synthase kinase beta (GSK3β) and the mitogen-activated protein kinase (MAPK) signaling pathway can replace some of the reprogramming factors during iPS cell generation [7], and these inhibitors can similarly stabilize the LIF/serum-dependent pluripotent state in blastocyst-derived stem cells or iPS cells from the the non-permissive NOD mouse strain [4], [8], [9], [10]. Thus, it appears that the LIF-dependent pluripotent state is metastable in NOD mice, meaning it is dependent on either the constitutive expression of ectopic reprogramming factors or the presence of small molecule inhibitors of the GSK3β and/or the MEK/ERK signaling pathway. In the absence of these exogenous factors, NOD iPS cells assume a stable EpiSC-like state, even when LIF is present in the culture media.

Genetic background appears to play an important role in stabilizing the LIF-dependent pluripotent state, yet its role in defining the FGF-dependent pluripotent stateis less clear. We explored the possibility of generating EpiSCs by iPS reprogramming of murine embryonic fibroblasts from the permissive129 and/or BL6 mouse strains in EpiSC culture conditions.

Unexpectedly, we found that even in the presence of EpiSC culture conditions, iPS cells adopt a naive ICM/ES-like pluripotent state. Thus, it appears that strain-specific genetic elements dictate that in permissive mouse strains, the ES-like pluripotent state is dominant following iPS reprogramming

## Results

### Generation and molecular analysis of FGF-iPSCs

**Figure 1:**
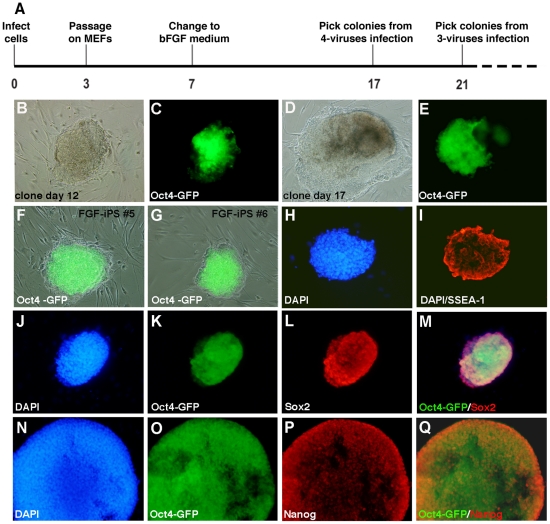
Generation of FGF-iPS from mouse fibroblasts by retroviral transduction. Schematic diagram illustrating a timetable of the critical steps of the reprogramming protocol utilized. Embryonic mouse fibroblasts were transduced with retroviruses carrying Oct4, Sox2 Klf4 and c-Myc and then transferred onto MEF feeder layers in DMEM medium supplemented with 10% FBS. After 4 days the medium was switched to a human ESC culture medium. Colonies activated the Oct4-GFP transgene after twelve days in culture (B–C) and are isolated for expansion after 17–18 days (D–E). After picking, single clones give rise to stable cell lines of FGF-iPSCs, which grow as round compacted colonies (F, H) expressing the Oct4-GFP transgene (G, I). Colonies expressing the Oct4-GFP (K, O, S) result positive for alkaline phosphatase activity (AP) (J), and SSEA-1 (M), Sox2 (P) and Nanog (T) immunoistochemistry. Merged figures for Sox2/Oct4-GFP and Nanog/Oct4- immunostainings are showed in Q and U, respectively. DAPI fluorescent staining is labeling cell nuclei (blue) in L, N, R.

Murine embryonic fibroblasts (MEFs) of E14 Oct4-GFP (BL6/tgOct4-GFP) were transduced with a cocktail of retroviruses expressing the iPS reprogramming factors (Oct4, Sox2, Klf4 and c-Myc) as shjown schematically in (Figure 1A). Upon transduction, the cells were passaged with trypsin and then re-plated onto a feeder layer of mitotically inactivated MEFs. From day 7 onwards, infected fibroblasts were maintained in bFGF medium (DMEM, 20% Serum Replacement and 4 ng/ml bFGF). Starting from days 10–12, we observed the emergence of tightly compact colonies, which had reactivated the Oct4-GFP transgene (Figures 1B–1E). On day 17, single colonies were picked, and further propagated in bFGF medium. Unexpectedly, upon subsequent passaging, the cultures uniformly maintained a characteristic murine ES-like morphology, with round and compacted cell clusters expressing Oct4-GFP (Figures 1F–1I) which contrasts sharply with the flattened two-dimensional colony morphology of EpiSCs derived and maintained under the same culture conditions. We name these cells mouse FGF-iPSCs, to distinguish them from conventional LIF-dependent murine ESCs and iPSCs. In addition to the ES-like morphology, FGF-iPSCs cultures exhibited homogeneous SSEA-1, but not SSEA-3, SSEA-4, TRA-1-60 and TRA-1-81 expression (Fig. 1H, I). In addition, FGF-iPSCs reactivate endogenous Oct4-GFP, Sox2 and Nanog (Fig. 1J–Q, Figure S1). Cytogenetic analysis of two independent FGF-iPS cell lines revealed a normal karyotype (2n = 40) even after prolonged culture at high passage number (passage 28, 3 months in culture) (Fig. S1C and data not shown). As expected, bisulfite sequencing demonstrated hypomethylation of the Oct4 promoter region as tested in 12 different CpG islands scattered around 350 bp of the Oct4 minimal promoter (Fig. S1A). Correct establishment of the reprogrammed cell state was confirmed by complete silencing of the exogenous reprogramming factors as revealed by qPCR (Figure S1B).

### Growth factor culture conditions affect the dynamic of iPS reprogramming

**Figure 2:**
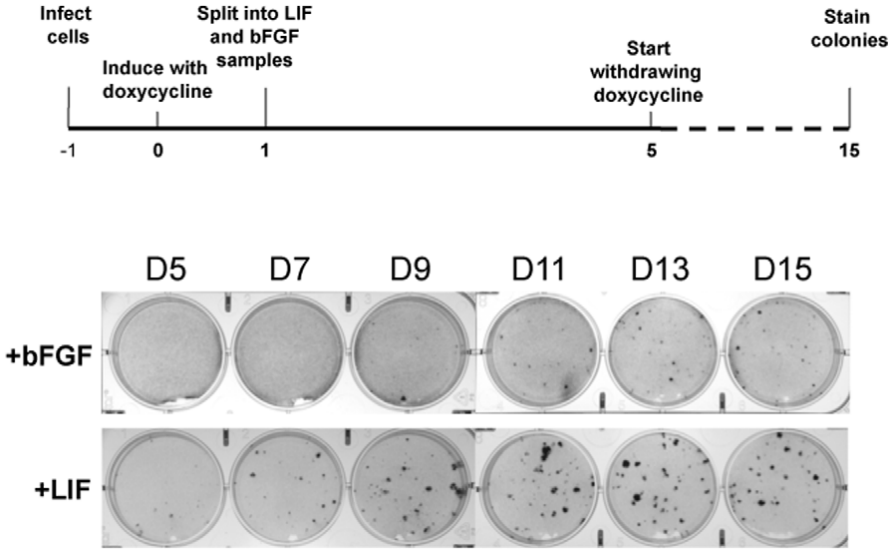
Derivation of murine iPS cells in the presence of bFGF by inducible lentiviral transduction. (A) Schematic representation of the strategy used for the reprogramming of murine fibroblasts into iPS cells in the presence of LIF or bFGF. (B) Image of an EpiSC-like iPS cell colony derived in bFGF. (C) Schematic of the temporal analysis of the emergence of stable iPS colonies in the presence of bFGF. At day -1, murine embryonic fibroblasts are infected with the doxycycline-inducible reprogramming factors and rtTA. AT day 0, doxycycline is added to the culture media to initiate expression of the ectopic reprogramming factors. On day 1, the sample is split into dishes which are subsequently maintained in the presence of LIF or bFGF. Starting at day 5, doxycycline is withdrawn at set time intervals and emergence of stable, factor-independent colonies is analyzed at day 18. (D) Emergence of stable, factor independent colonies upon iPS reprogramming in the presence of bFGF (top panel) or LIF (bottom panel). Indicated on top is the day at which doxycycline was withdrawn from the culture to silence the ectopic reprogramming factors.

To examine the effect of the growth factor conditions on the dynamic of the iPS reprogramming response, we reprogrammed from 129/BL6 F1 embryonic fibroblasts either in the presence of LIF/serum (standard murine ES/iPS conditions) or in the presence of bFGF (EpiSC conditions). For this purpose, we employed the recently reported STEMCCA inducible lentiviral vector system, which allows the expression of the four reprogramming factors (Oct4, Sox2, Klf4 and cMyc) from a single lentiviral vector in a doxycyclin-inducible manner through the action of the reverse tetracycline transactivator (rtTA) at high efficiency (Figure S2A) [11]. Figure 2A schematically displays the experimental setup. Murine embryonic fibroblasts were transduced with the doxycyclin-inducible reprogramming factors and rtTA and reprogramming was induced 24 hours after infection (t = 0). At day 1, the sample was split and cells were cultured either in the presence of LIF (mES conditions) or in the presence of bFGF (EpiSC conditions). At set time intervals (between day 5 and day 15), ectopic reprogramming factors were silenced by removal of doxycycline. Colonies were visualized by Crystal Violet staining on day 18. After approximately 10–12 days, iPS colonies appeared under both conditions, and the LIF-derived iPS cells displayed a characteristic ES-like colony morphology, whereas we noted iPS cells derived in the presence of bFGF displayed the characteristic flattened colony morphology of EpiSCs (Figure S2B). However, the EpiSC-like colonies were unstable, and upon withdrawal of the ectopic reprogramming factors, most of the EpiSC-like iPS cells assumed a fibroblast-like morphology, indicating that they were partially reprogrammed and had not activated their endogenous pluripotency program. Indeed, the Oct4-GFP reporter gene present in these cells was not reactivated in the EpiSC-like iPS cells, whereas the control iPS cells did reactivate Oct4-GFP (not shown). However, in the FGF conditions, few colonies remained after silencing of ectopic reprogramming factors, which could be stably propagated in the presence of bFGF, and yet displayed the characteristic murine ES-like colony morphology. The number of stable FGF-iPS colonies increased with longer reprogramming time, but lagged behind compared to the LIF control iPS cells. As shown in figure 2B, in the presence of bFGF, stable iPS colonies emerged after 9 days of doxycycline-induced reprogramming, whereas in the presence of LIF, stable colonies were noted 4 days earlier.

Thus, it appears that while the culture growth factor conditions affect the dynamic of the iPS reprogramming process, with stable colonies emerging late under FGF growth factor conditions, the general outcome of the reprogramming response is not affected by the culture conditions.

### FGF-iPSCs display molecular and epigenetic features of the ICM/ES cell pluripotent state

**Figure 3:**
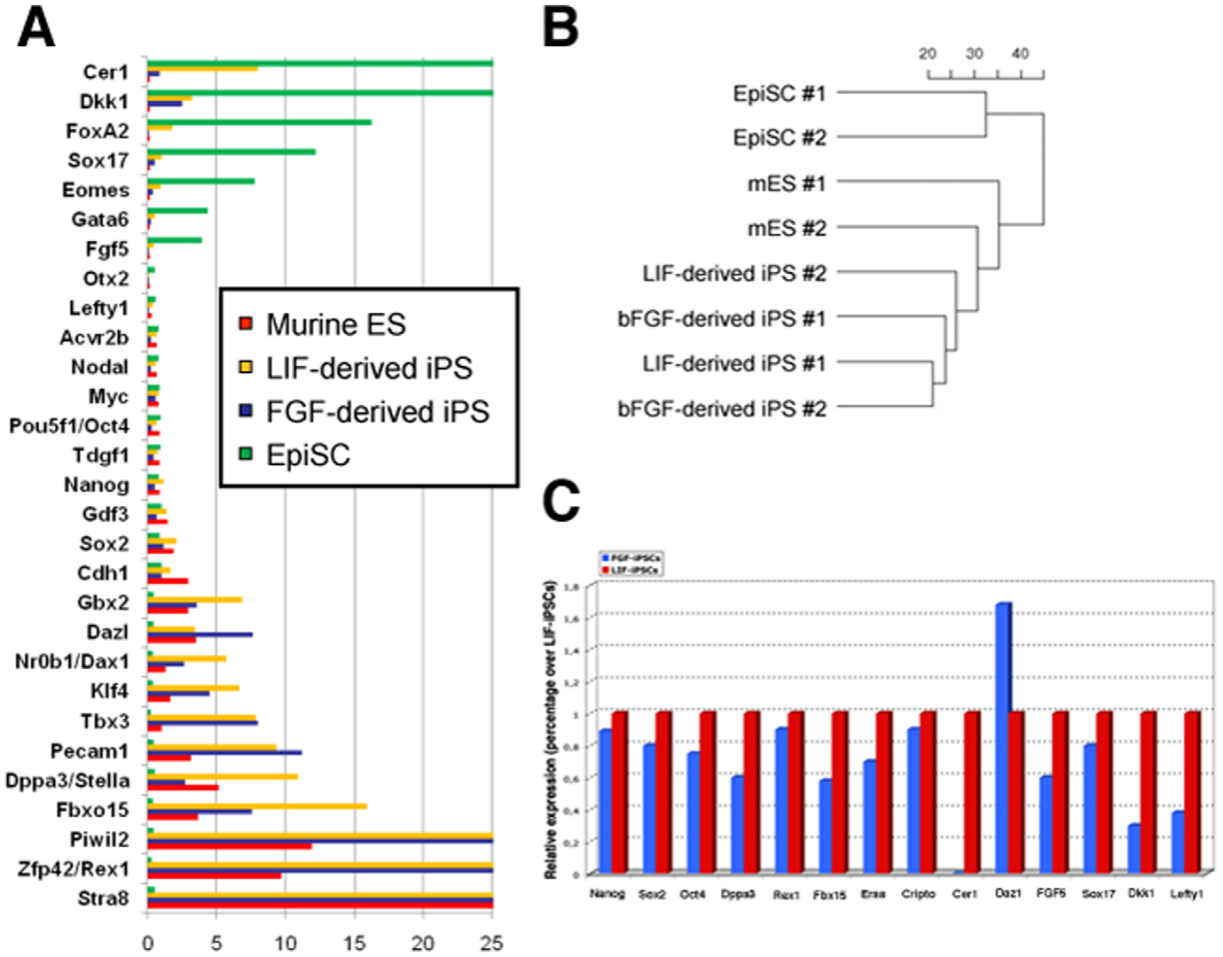
The ES-like pluripotent state is dominant in the 129/BL6 genetic background. (A) Comparative gene expression analysis among conventional murine ESCs, LIF-iPSCs, FGF-iPSCs and EpiSCs for candidate genes of pluripotency. Normalized expression intensity values (scaled median ratio) were obtained from Agilent whole-genome microarrays. Two biological replicates were used for all cell types. (B) Unbiased cluster analysis of global gene expression profiles of two independent FGF-derived iPS clones, two independent LIF-derived-iPS clones, two murine ES cell lines and two EpiSC cell lines. (C) qPCR quantification of the pluripotency associated genes Nanog, Sox2, Oct4, Rex1, Fbx15, Eras, Cripto, Cer1, Daz1, Fgf5, Sox17, Dkk1 and Lefty1 in LIF-iPSCs compared to FGF-iPSCs.

The emergence of iPS cell colonies with typical murine ES-like characteristics under EpiSC culture conditions was unexpected and hence we performed genome-wide expression analysis to further characterize these cells. As shown in Figure 3A, FGF-iPSCc display a gene expression pattern characteristic of murine ES cells, including the inner cell mass markers Rex1, Nanog, Oct4, Sox2, Sall4, Gdf3 and Eras. In contrast, typical EpiSC markers, including FGF5, Eomes (also known as Tbr2), FoxA2 and Cer1 were not expressed in FGF-iPSCs (Fig. 3A). Microarray data were confirmed by qPCR expression analysis (the list of used primers is available in table S1) (Fig. 3C). Hierarchical cluster analysis of the global gene expression profiles of FGF-iPSCs cells, LIF-derived iPS cells, murine ESCs and EpiSCs revealed that FGF-iPSCs are highly similar to murine ES- and LIF-derived iPS cells, whereas EpiSCs cells form a separate cluster of unrelated cells (Fig. 3B). Starting fibroblasts are absent in this analysis as most of the investigated were not expressed in the cells prior to iPSC reprogramming.

**Figure 4:**
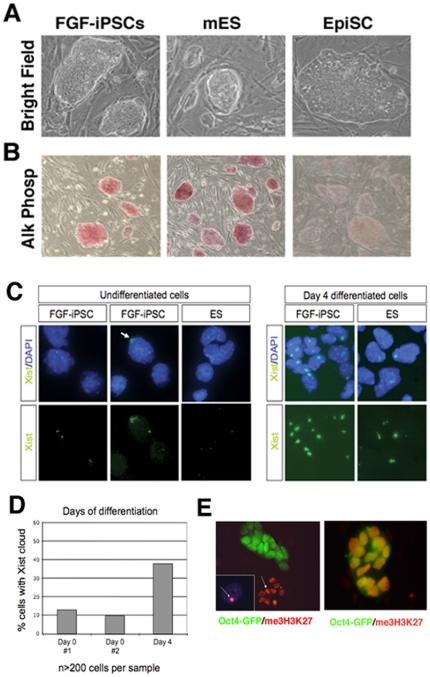
FGF-iPSCs do not share crucial properties of EpiSCs. (A) Colony morphology of FGF iPSCs (left), murine ES cells (middle) and EpiSCs (right). Note the dome-shaped, three dimensional morphology of the FGF-iPS cells. (B) Alkaline phosphatase (AP) staining of the cells in (A). FGF-iPSCs are expressing AP at levels comparable with murine ESCs. On the contrary EpiSCs are negative for this markers. (C) FISH analysis show that the majority of FGF-iPSCs contains two active X chromosomes. FGF-iPSCs robustly display X-inactivation upon differentiation. The percentage of FGF-iPSCs containing a Xist-cloud is quantified. (D) Approximately 90% of the undifferentiated FGF-iPSCs contain two active X-chromosomes, whereas 40% of the FGF-iPSCs display X-inactivation after 4 days of differentiation. (E) Double immunostaining for me3H3K27 and Oct4-GFP in two female FGF-iPS cell lines. Differentiated cells which lost Oct4 expression show an evident me3H3K27 positive nuclear staining corresponding to the inactivated X-chromosome (arrow). On contrary, undifferentiated Oct4-GFP+ cells do not present any me3H3K27 positive nuclear puncta. White arrow indicates the diagnostic staining for the inactive X chromosome. Inset in D is a higher magnification of a single cell nucleus with a me3H3K27 positive inactivated X-chr.

Alkaline phosphatase (AP) is a widely used marker distinguishing murine ESCs, which are expressing AP, from EpiSCs, which are negative for this marker. Interestingly, iPSCs derived in the presence of bFGF were strongly positive for the AP staining, further confirming their similarity to ESCs (Fig. 4A, B).

In addition to the above molecular and morphological characteristics, we examined the epigenetic properties of the FGF-iPSCs. The pluripotency mediator Oct4 is differentially expressed from two distinct enhancer regions; a distal enhancer which drives Oct4 expression in murine ES cells and and a proximal enhancer which mediates Oct4 expression in EpiSCs [12]. Thus, Oct4 enhancer choice is a distinctive feature between ES cells and EpiSCs. As shown in Figure S1D, Oct4 expression is driven by the ES-specific distal enhancer in FGF-derived iPS cells, as well as the ES- and LIF-derived iPS controls. In contrast, as expected, the proximal enhancer is active in control EpiSCs.

In addition, we examined the X-inactivation state of iPSC clones from a female cell line by RNA FISH for Xist. As demonstrated in Figure 4C–D the majority of FGF-iPSCs contains two active X chromosomes as demonstrated by the presence of only basal (pinpoint) Xist expression on both X chomosomes as also observed in the mESC control cells (Figure 4C) whereas in some cells an Xist-cloud was observed (Figure 4C, arrow). As expected, FGF-iPSCs robustly display X-inactivation upon differentiation, demonstrating that the cells are capable of X-inactivation (Figure 4C). The percentage of FGF-iPSCs containing a Xist-cloud is quantified in Figure 4D and demonstrated that approximately 90% of the undifferentiated FGF-iPSCs contain two active X-chromosomes, whereas 40% of the FGF-iPSCs display X-inactivation after 4 days of differentiation. The percentage of FGF-iPSCs displaying an Xist-cloud (10%) is higher than X-inactivation observed in control mESCs (0.5%) and is perhaps reminiscent of the higher percentage of X-inactivation also observed in human ESCs. Finally, immunofluorescence-based detection of the trimethylated H3 lysine 27 (me3H3K27), a repressive histone modification, revealed the absence of a silent X chromosome in two undifferentiated female FGF-iPS cell lines (Fig. 4E). This is in stark contrast to EpiSCs which exhibit complete X-chromosome inactivation similar to their tissue of origin. Together these data demonstrate that in addition to morphological and molecular similarities, FGF-iPSCs display an epigenetic profile characteristic of mESCs as well.

### Murine FGF-iPSCs are FGF-dependent

**Figure 5:**
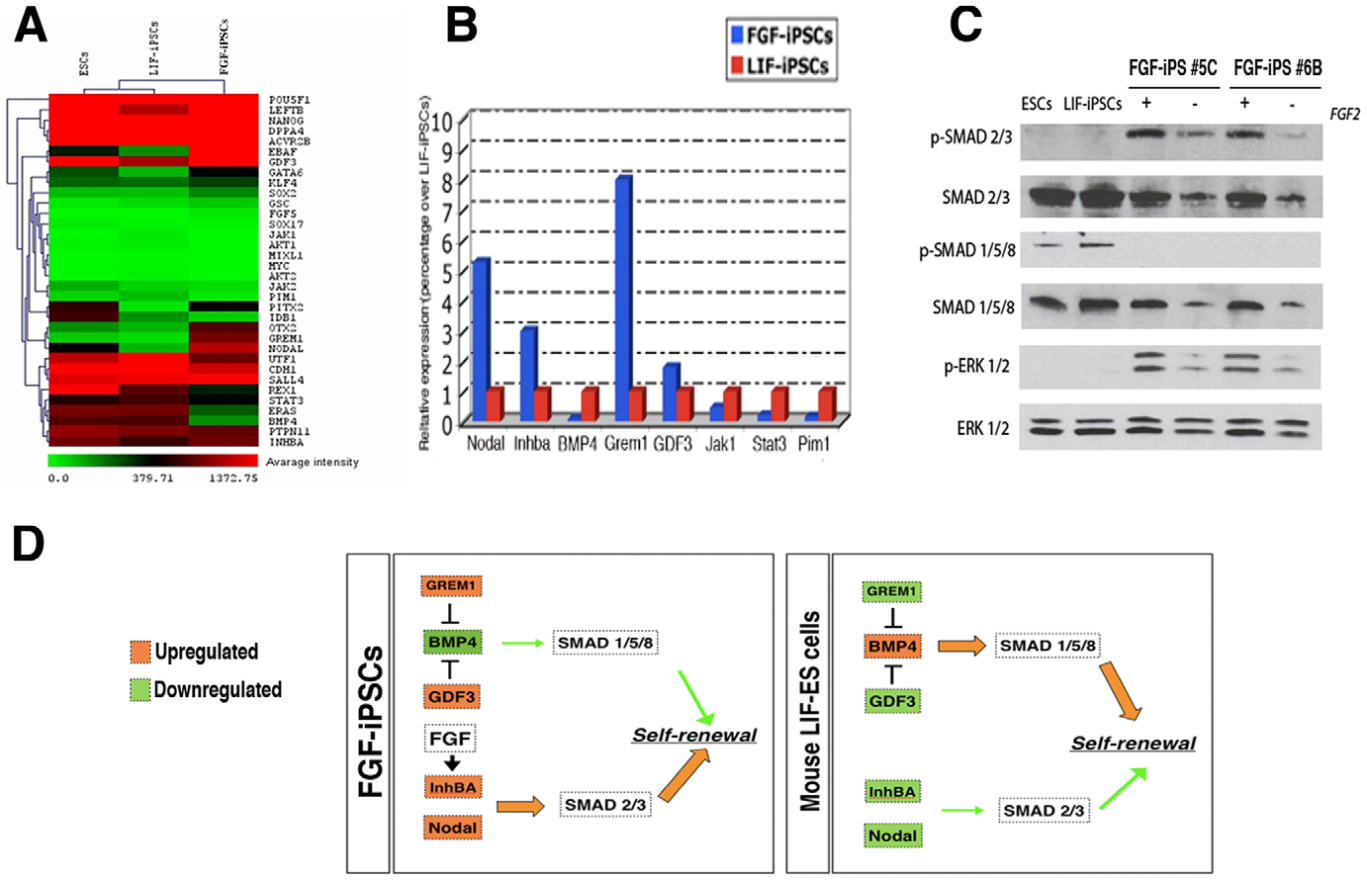
FGF-iPSCs show activation of opposite TGFb and FGF intracellular pathways with respect to ESCs and LIF-iPSCs. (A) Heat-map diagram of a small selection of self-renewal related genes that are in common between the three cell types. Shown are genes which are part of the pluripotency machinery, primitive-streak, Jak-Stat pathway and TGFb signaling pathway. (B) qPCR analysis for mRNA level quantification of the Nodal, Inhba, Bmp4, Gremlin-1 (Grem1) and Gdf3 genes in FSR-iPSCs and ESCs. (C) Immunoblotting showing the phosphorylation levels of the different SMAD and ERK1/2 proteins and compared among ESCs, LIF-iPSCs and FSR-iPSCs, tested as protein extracts of cell cultures grown in their normal conditions. In some conditions FGF-2 (bFGF) was withdrawn for six days. (D) General outline illustrating the state of activation of the two different molecular branches of the TGFb signaling and their key molecular components in FSR-iPSCs and LIF dependent stem cells (ESCs and LIF-iPSCs).

Despite the common expression of pluripotency genes between LIF or FGF derived iPSCs, important differences emerged in the expression levels of genes encoding key factors of the Nodal/Activin or Jak/Stat3 pathways between the two cell types. In fact, FGF-iPSCs exhibited high expression levels of Nodal and Inhba and, simultaneously, a low expression of genes downstream of the LIF-JAK-STAT3 signalling pathway (Stat3, Jak1, Jak2 and Pim1) in comparison to conventional ESCs and iPSCs as detected by microarray profiling and confirmed by qPCR analysis (Fig. 5A, B).

**Figure 6:**
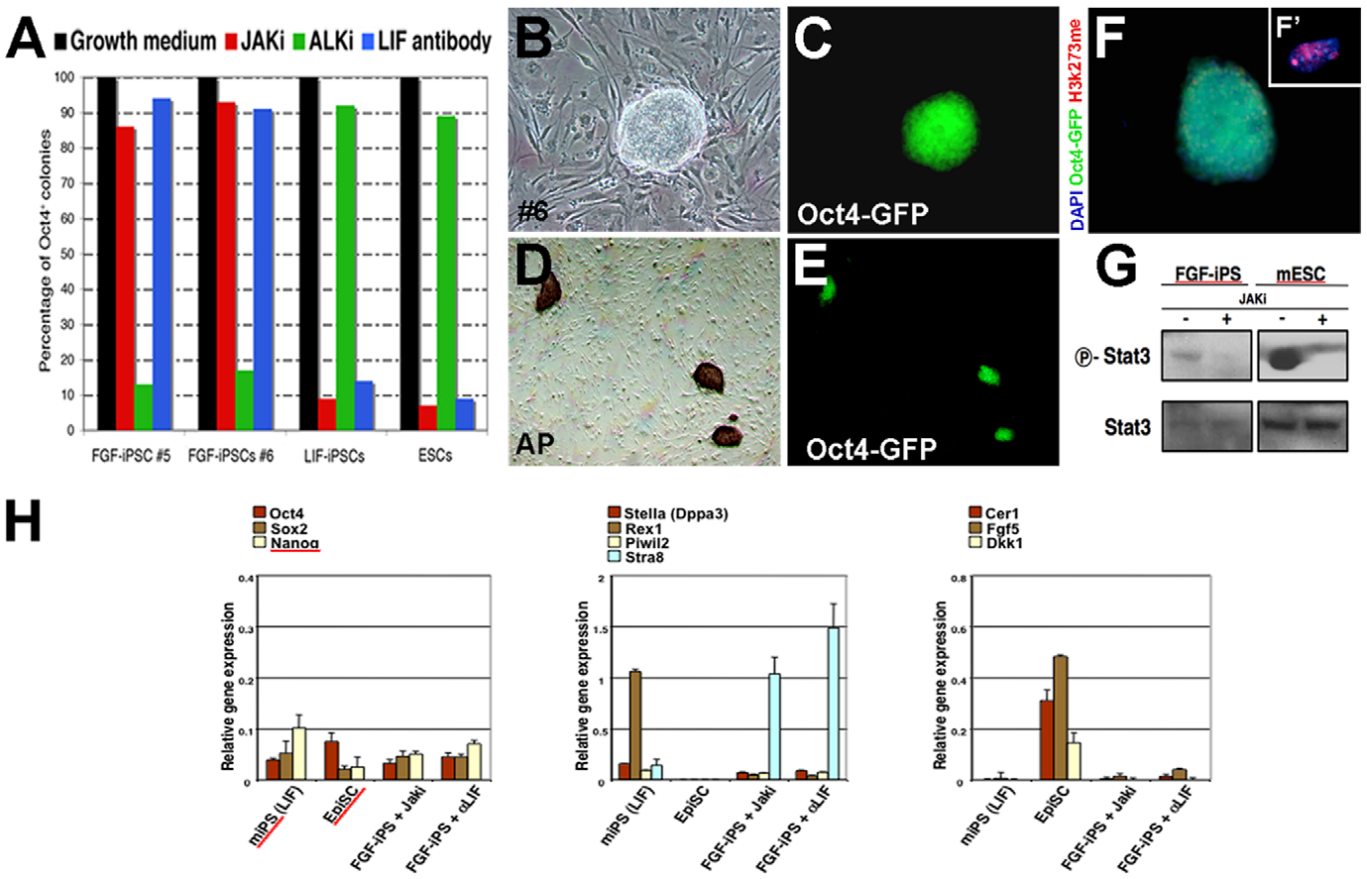
Effect of Jaki, ALKi and LIF-antibody on FGF-iPSCs and LIF dependent ES and iPS cells. (A) All cell samples were plated onto inactivated MEFs and treated for one week with Jaki, Alki or LIF-blocking antibody to evaluate the dependency from LIF/Stat pathway or TGFb pathway. The percentage of persisting Oct4-GFP positive colonies was evaluated at day 7. Reported values were normalized over the internal control (number of colonies in growth medium without inhibitors, black bars). The number of cell colonies are represented on the Y axis. After treatment with Jaki for 15 days the FGF-iPSCs maintein the morphology (B) and the Oct4-GFP expression (C). (D-E) AP staining reveal the mainteinance of positive colonies in treated cells. (F) After treatment the cells show no X chromosome inactivation. (G) Western blot show the expression of Stat3 and P-Stat3 in FGF-iPSCs and mESC. Stat3 phosphorylation is suppressed by Jaki. (H) Gene expression analysis by qPCR among mESCs, FGF-iPSCs, EpiSCs, FGF-iPSCs + Jaki and FGF-iPSCs + alphaLIF.

To confirm that FGF-iPS are maintained independent of JAK-STAT3 signaling, we cultured FGF-iPSCs in the presence of a JAK inhibitor (JAKi) or a LIF blocking antibody, in order to inhibit Stat3 phosphorylation (Fig. 6A). As shown in Figure 6G, addition of the JAKi inhibitor efficiently eliminates STAT3 phosphorylation under these conditions both in FGF-iPS and conventional mESCs, in which STAT3 is robustly activated. FGF-iPSCs could be propagated for more than 7 passages in the presence of JAKi inhibitor while maintaining their undifferentiated state and Oct4-GFP endogenous expression (Fig. 6B, C). In contrast, we observed rapid loss of pluripotency gene expression when conventional mouse ESC and/or iPSC were cultured under the same conditions (Fig.6). Furthermore, these cells displayed a strong AP activity and lacked any evident me3H3K27 staining ruling out the induction of Epi-like stem cells in these conditions (Fig. 6D–F). Accordingly, FGF-iPSCs maintained for 5 passages in the presence of JAKi inhibitor, retained their characteristic ESC-like gene expression profile with expression of ESC-like markers Stra8, Rex1 and Stella (Dppa3) and absence of epiblast marker expression (Cer1, Dkk1 and FGF5) (Fig. 6H). Conversely, inhibition of TGFbeta/Activin signaling using a specific inhibitor of the type I Activin receptor (ALK-I) resulted in rapid FGF-iPSC differentiation, while this inhibitor did not affect mESC self-renewal (Fig. 6). Control EpiSCs and human ESCs similarly differentiated upon ALK-1 inhibition (data not shown). In addition, FGF withdrawal or FGF receptor inhibition by the application of SU5402 in FGF-iPSCs for six days resulted in widespread cell death (Figure S3). These findings demonstrate that FGF-iPSCs are maintained independent of the activation of the JAK-Stat3 signalling pathway. Instead, FGF-iPSC self-renewal relies on the continued presence of FGF stimulation and activity of the TGFbeta/Activin signaling cascade.

Indeed, downstream mediators of FGF- and TGFbeta/Activin signaling are activated in FGF-iPSCs., We assessed Smad 2/3, Smad 1/5/8 levels in mouse ESCs, LIF-iPSCs and FGF-iPSCs by Western blot analysis. ESCs and LIF-iPSCs showed phosphorylation of Smad 1/5/8, indicating active transduction of Bmp signaling (Fig. 5C). On the contrary, two independent FGF-iPSC lines displayed a strong activation of Smad 2/3 concomitant with undetectable levels of Smad 1/5/8 active forms (Fig. 5C). qPCR analysis confirmed that specific to FGF-iPSCs, Bmp4 expression was significantly down-regulated together with the up-regulation of Gdf3 and Gremlin-1 (Grem1), two well-known Bmp4 antagonists [13], [14], [15] (Fig. 5B). Taken together, these findings demonstrate that self-renewing FGF-iPSCs exhibit activation of FGF and TGFbeta/Activin downstream signaling pathways and undetectable BMP4 signalling, in contrast to mESCs, in which the BMP4 signalling pathway is prominently activated and TGFbeta/Activin signalling is low (Fig. 5D).

**Figure 7:**
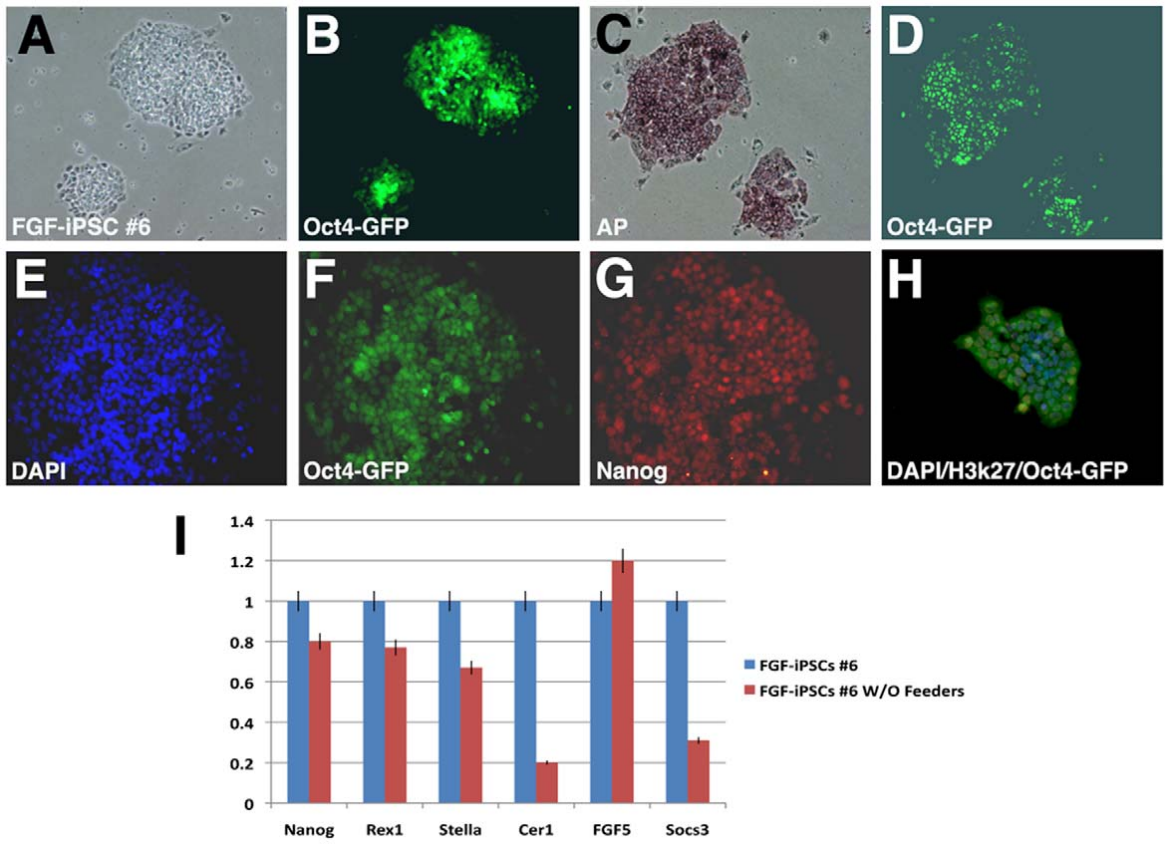
Feeder-free culture of FGF-iPSCs. The FGF-iPSCs growth in the absence of feeder layer as colonies expressing Oct4-GFP (A–B). (C–D) The colonies are positive for alkaline phophatase. (E–G) FGF-iPSCs maintain the expression of Nanog in a feeder free condition. (H) Staining for H3K27 on FGF-iPS colonies show the absence of X-chromosome inactivation.

Next, to definitively exclude the role of feeder cells in promoting FGF-iPS stem cell properties, we serially cultured FGF-iPSCs on fibronectin-coated plates in the absence of fibroblast feeder cells. At passage 6, corresponding to 5 weeks of culture in these conditions, FGF-iPS colonies revealed strong Oct4-GFP and Nanog endogenous expression as well as evident AP activity (Fig. 7A–G). In contrast, FGF-iPSCs did not show inactivation of the X-chromosome as indicated by lack of me3H3K27 staining (Fig. 7H). In line with these findings, FGF-iPSCs expressed Nanog, Rex1 and Stella (Dppa3) at similar levels to those detected when cultured on feeder conditions, and the EpiSC markers Cer1 and FGF5 were not found up-regulated as tested by qPCR (Fig. 7I). Interestingly, expression of the STAT3-induced gene Socs3 was strongly reduced suggesting that this signaling is generally repressed in these culture conditions (Fig. 6I). Thus, FGF-iPSCs conserved those cardinal molecular and epigenetic features closely associated to pluripotency even when deprived of feeder layers for prolonged time.

To test the influence of the growth factor milieu on pluripotency of FGF-iPS cells, we examined the effect of LIF stimulation on these cells. Upon culture for 10 days in a traditional mouse ESC culture medium (20% Serum, LIF), the vast majority of FGF-iPSCs were rapidly induced to differentiate causing the fragmentation of the colonies into numerous polygonal-shaped GFP- isolated cells (data not shown). However, few cells tightly adherent in small colonies maintained a strong Oct4-GFP expression. Upon trypsinization into single cells and propagation on MEFs, these cells organized into typical mouse ESCs colonies, a morphology maintained even after extensive expansion (P10, 6 weeks in culture) (Figure S4A–D). We termed these cells “LIF stimulated FGF-iPSCs” to indicate their FGF-iPSC origin. The conversion efficiency was approximately ∼0.01% comparable process to the recently reported conversion of EpiSCs into mESC-like cells [16]. Furthermore, when culture conditions were switched back to the original FGF culture medium, the cells re-acquired all the FGF-iPSC morphological characteristics (Figure S4E, F). These results emphasize once again that FGF-iPS cells do not depend on LIF signals for their continued self-renewal, but instead differentiate when switched to LIF culture conditions. However, similar to the recently reported conversion of EpiSCs into mESC-like cells, a small fraction of FGF-iPSCs can adapt to the LIF culture conditions and convert into a mESC-like state.

### Murine FGF-iPSCs generate chimeras with germline transmission

**Figure 8:**
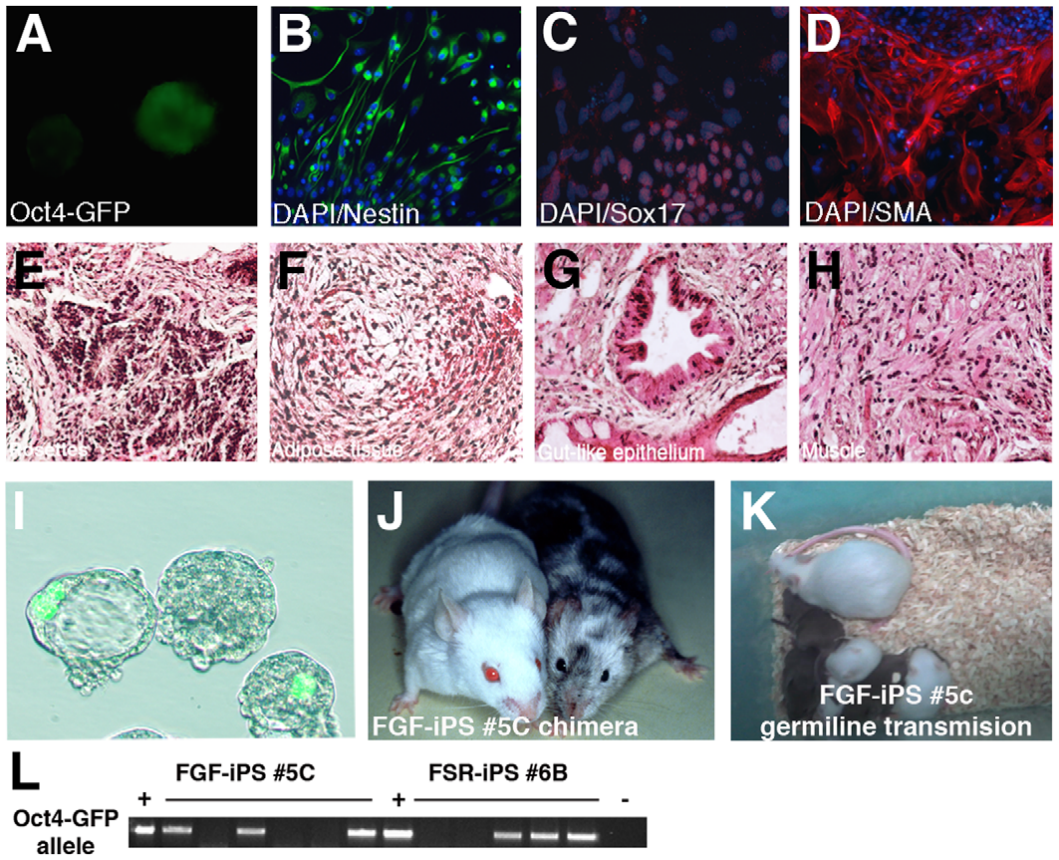
*In vitro* and *in vivo* pluripotency of FGF-iPSCs. (A) FGF-iPSCs were detached with collagenase IV from the substrate to induce embryoid body (EB) formation. After 5 days in culture, the majority of EBs downregulate the GFP transgene. (B) EBs plated in N2-medium generate nestin positive neural progenitor cells as highlighted by immunofluorescence with its specific antibody. (C) EBs were plated on gelatin coated dishes containing DMEM medium supplemented with 20% FBS. After 20 days in such condition, islands of beating cardiomyocite became evident (see Movie S1). Cell differentiation was confirmed by immunoistochemistry for the mesodermal marker smooth muscle actin (Sma) (C) and for the endodermal marker Sox17 (D). (E–H) Ematoxylin and Eosin histological staining of teratomas, derived by subcutaneously injection of 1 million FGF-iPSCs, showed cartilage (E), adipose tissue (F), gut-like epithelium (G) and muscle (H). (I) Oct4-GFP positive FGF-iPSCs integrate into the inner cell mass of mouse blastocyst after morula aggregation and contribute, thereafter, to viable highly chimaeric animals (on the right) as compared to unmanipulated mice (on the left) (J). (K) Germiline contribution of FGF-iPS #5 and #9 chimera. (L) Genotyping demontrate the presence of the Oct4-GFP transgene in the offspring.

To determine the developmental potential of FGF-iPSCs, we examined their *in vitro* and *in vivo* differentiation. We generated aggregates, termed embryoid bodies (EBs), in which pluripotent stem cells differentiate in a manner closely resembling early embryonic development, with the formation of early derivatives of the three embryonic germ layers and downregulation of pluripotency genes. Indeed, we observed rapid loss of Oct4-GFP expression in FGF-iPSC-derived EBs after 4 days of differentiation (Fig. 8A). EBs plated onto matrigel coated dishes in serum-free medium containing bFGF differentiated into Nestin expressing neuronal cells (ectoderm) (Fig. 8B). When these EBs were incubated on gelatin-coated tissue culture plates in DMEM medium supplemented with 10% FBS for 15 to 20 days, they differentiated into a wide range of cell types including Sox17 positive endoderm progenitors and Sma positive smooth-muscle cells (mesoderm) (Fig. 8C, D).

To test pluripotency *in vivo*, FGF-iPSCs were injected subcutaneously into nude mice. Six weeks after transplantation, teratomas were isolated and histological analysis confirmed the presence of well defined differentiated derivatives of the three embryonic germ layers, including neural tissue, adipose tissue, epithelial structures and muscle fibers (Fig. 8E–H).

The most important functional difference between mESCs and EpiSCs is the striking inability of EpiSCs to form chimeras upon morula or blastocyst injection [1], [2]. We examined the ability of FGF-iPS cells to integrate into preimplantation stage mouse embryos by aggregating mouse embryos at the 8 cells or morula stage with clumps of FGF-iPSC. GFP-positive FGF-iPSCs readily integrated with the developing inner cell mass and chimeric animals were successfully obtained at a frequency of approximately 24% (11 chimeras out of 46 animals) with a coat-color contribution ranging between 5% to 80% (Fig. 7I,L). Furthermore, germline transmission was obtained from two chimeras, derived from two independent FGF-iPSC lines (#5 and #9), as revealed by coat color and confirmed by the presence of the Oct4-GFP transgene in the offspring (Fig. 8M, N). These results demonstrate that the developmental potential of FGF-iPSCs is comparable to that normally exhibited by conventional LIF-dependent ESCs and iPSCs and FGF-iPSCs can functionally contribute to chimera formation.

We did not test the ability of FGF-iPSCs to generate entirely iPSC-derived mice by tetraploid blastocyst complementation, but analysis of the Dlk1-Dio locus revealed that in at least one FGF-iPSC clone the expression of Gtl2 was correct (Figure S5), demonstrating that similar to LIF-dependent iPSCs, the imprint status of this locus is not always preserved, but some clones can be identified that show a correct imprint status.

## Discussion

Together, our data demonstrate that derivation of stable iPS cells in the presence of bFGF yields two types of colonies. Colonies with morphological characteristics of EpiSCs, which are unstable and remain dependent on the constitutive expression of ectopic reprogramming factors. These are likely partially reprogrammed colonies, since they fail to reactivate endogenous pluripotency genes. In addition, stable, ectopic factor-independent colonies emerge, which display morphological, molecular, epigenetic and functional properties of murine ES cells. These murine FGF-iPSCs are maintained in an FGF-dependent fashion (>40 passages) with a normal karyotype, and display multilineage differentiation *in vitro* and broad developmental potential *in vivo*, including the generation of germline competent chimeras.

Together our results demonstrate that while the growth factor conditions influence the dynamic of the somatic cell reprogramming response, the ES-like pluripotent state is the dominant endpoint that is achieved independent of the culture growth factor conditions. Several lines of evidence make it highly unlikely that the ES cell pluripotent state is the result of low-level residual LIF activity emerging from the MEF feeders. First, FGF-iPS cells can be maintained under defined culture conditions in the absence of MEF feeders. Second, the FGF-derived iPS cells are dependent on bFGF signaling for their continued self-renewal, and are not affected by prolonged inhibition of JAK-STAT signaling. Finally, switching the cells to conventional mES culture conditions with addition of LIF results in FGF-iPSC differentiation, indicating that LIF is in fact incapable of maintaining FGF-iPS cells.

FGF-iPSCs and standard ESCs or iPSCs do not represent alternative metastable cell states as described for ESCs and EpiSCs, but cells with similar properties sharing an equivalent pluripotency state. Consistent with this, while EpiSCs require the stable exogenous expression of Klf4 or c-Myc for their conversion into conventional ESCs or, alternatively, the presence of small molecules that can replace these factors during reprogramming [4], [17], FGF-iPSCs can be converted to conventional LIF-dependent iPSCs by simply switching growth factor culture conditions. A similar approach was shown to efficiently induce the conversion of epiblast cells from the primitive ectoderm into ES-cell-like cells (rESCs) [16]. Using an ESC-specific Oct4 distal enhancer reporter cell line, Bao and colleagues demonstrated that approx. 22–36% of epiblast cells respond to a switch in growth factors from FGF to LIF by upregulating the ESC-specific GFP reporter [16]. This efficiency in rESC derivation is much higher than the conversion rate we observed between FGF-iPSCs and LIF-dependent ESCs, indicating a relative more robust epigenetic barrier exists between these two cell types. However, when the same switch was applied to established EpiSC, the efficiency for generating rESCs was extremely more reduced and therefore more similar to what described in our experimental conditions.

Iinhibition of Erk signalling, either by BMP4-induced upregulation of ID proteins or using small molecule inhibitors, is required in mESCs to maintain the ICM-like pluripotent state [18]. It is striking that FGF-dependent iPSCs maintain a naive pluripotent state in the presence of strong ERK activity. The morphological and epigenetic similarities between murine EpiSCs and human ESCs suggest that human ESCs, despite their blastocyst origin, exist in a primed pluripotent state. Our data now demonstrate that a naive pluripotent state can be achieved in FGF-dependent murine iPSCs. Revealing the molecular mechanisms that install a naive pluripotent state in FGF-iPSCs may provide important cues the identity of human ESCs.

Hanna and colleagues recently demonstrated that in the non-permissive NOD genetic background, the ES-like pluripotent state is metastable, and remains dependent on the ectopic expression of Klf4 or cMyc or the presence of small molecule inhibitors of GSK3β or the MEK-ERK signaling pathway. In this genetic background, the EpiSC pluripotent state appears dominantly stable and is achieved upon withdrawal of ectopic reprogramming factors. Our results demonstrate that the opposite is true for ‘permissive’ mouse strains such as the 129Sv, C57B/L6 and 129/BL6 F1 genetic backgrounds used in this study. The genetic elements that allow the derivation of ES-cell lines in these strains may be the same that dominantly stabilize the ES-like pluripotent state in iPS cells from these strains. The murine ES-like state offers several functional advantages over the epiblast-stem cell state, since it allows ready manipulation of the genome combined with the ability to generate chimeras. While gene targeting is commonplace in some mouse strains, other species do not readily allow gene manipulation using similar experimental approaches, since in most species the primed pluripotent state appears to prevail. Primed pluripotent stem cells are refractive to single cell culture, which severely hampers the clonal derivation of mutant cell lines. In addition, the murine ES-state allows the generation of chimeras and may thus facilitate the generation of animal mutants to model human disease in alternative species. Indeed, the recent derivation of rat pluripotent stem cells in a murine ES-like state illustrates this point [9], [10].

Understanding the nature of the genetic factors that dominantly stabilize the murine ES-like pluripotent state and uncovering their function in the stabilization of the ES-like pluripotent state is fundamentally important to understanding how to harness the power of this pluripotent state in future research and cell based therapies.

## Materials and Methods

### Ethics Statement

Animal care and experimental procedures were performed in accordance with “Decreto Legislativo n. 116 del 27 Gennaio 1992” and with the Institutional Committee for the Good Animal Experimentation of the San Raffaele Scientific Institute (IACUC 423).

This study was performed following ethical approval by the institutional review board of San Raffaele Scientific Institute.

### Culture of murine ES cells, EpiSCs and iPSCs

Murine ES (Di Stefano et al., 2009) and LIF-iPS cells (Di Stefano et al., 2009) were cultured on feeder layer in ES medium composed by knock-out DMEM medium (Invitrogen) containing 15% fetal bovine serum (Invitrogen), leukemia inhibitor factor (LIF) (Chemicon), 2-β-mercaptoethanol, L-glutamine and non-essential amino acids (Invitrogen) as detailed in Di Stefano et al. (2009). FGF-iPS cells were cultivated on mytomicin-inactivated MEF in human ESC medium containing knock-out DMEM medium (Invitrogen) containing 20% knock-out serum replacement (Invitrogen), 4 ng/ml bFGF (Peprotrech), 2-β-mercaptoethanol, L-glutamine and non-essential amino acids (Invitrogen). FGF-iPSCs were normally passaged in small clumps after treatment with collagenase IV (1 mg/ml) every 3 to 5 days in average. LIF-iPS, EpiSC and FGF-iPScells were cultured on Matrigel-coated dishes for two passages for depletion of feeder cells before DNA and RNA isolation. EpiSC [19] were maintained in the same conditions of FGF-iPS.

### Derivation of iPS cells using retroviral vectors

Oct4-GFP embryonic or post-natal fibroblasts were obtained from the OG2 transgenic mouse line kindly provided to us by Dr. Michele Boiani (Max Planck Institute, Münster, Germany). Briefly, E15.5 transgenic embryos were isolated, washed repetitively in PBS and then the heads and all the visceral tissues dissected out. The remaining carcasses were washed, minced and dissociated after a trypsin treatment of 30′ at 37°C. Single cells and small aggregates were cultured and propagated in DMEM medium (Invitrogen) with 10% FBS (Sigma). For post-natal fibroblasts, clipped tails were rinsed in PBS supplemented with antibiotics and careful minced, before digestion into trypsin for 20′ at 37°C. Digested tissue was finely disaggregated with Pasteur pipettes and single cell suspension plated and propagated in DMEM medium (Invitrogen) with 10% FBS (Sigma). For induction of FGF-iPS cells, 200.000 MEFs were seeded onto gelatin-coated dishes in DMEM medium with 10% serum and transduced by pLIB-based retroviruses for mouse Oct4, Klf4, Sox2 and c-Myc. Two days after, the cells were harvested and replated on mytomicin-inactivated MEF in 100 mm dish and incubated in human ESC medium. After 15–17 days (for O-S-K-M transduced cells) or 21–23 days (for O-S-K/O-S-M), the FGF-iPS colonies were picked for expansion on MEF feeder cells in human ESC medium. A schematic diagram of the reprogramming protocol used is shown in Figure 1A. Retroviral production was performed as described before [6]. Briefly, Murine cDNAs for Oct4, Klf4, Sox2 and c-Myc were amlified by PCR from ES cells cDNA and cloned into Moloney-based retroviral vector pLIB (kindly donated by Dr. Marius Wernig, Stanford University, CA). Plat-E cells were plated at 4×10^6^ cells per 100 mm dish and incubated overnight. Cells were transfected with 10 µg of vector, according to a conventional CaCl_2_ transfection protocol. After 30 h, medium was collected, filtered through 0.44 µm cellulose acetate filters and supplemented with 4 µg/ml Polybrene (Sigma). MEFs were then exposed to viral-polybrene containing supernatants.

### Derivation of iPS cells using STEMCCA vectors

Fibroblasts were harvested from tails of Oct4-GFP/R26-M2rtTA transgenic newborn mice and maintained in DMEM with 10% FBS, 2 mM L-Glutamine and Pen/Strep (all Invitrogen). To produce infectious viral particles, 293 T cells cultured on 10 cm dishes were transfected with the STEMCCA vectors together with the packaging plasmids VSV-G and Δ8.9 using Lipofectamine2000 (Invitrogen). The fibroblasts were transduced at ∼4000 cells/cm^2^ on 10 cm TC dishes with concentrated virus. The transgenes were induced the following day by adding 2 ug/ml Doxycyline (Sigma) to the media. After one more day cells were split onto two new 10 cm dishes and the media was changed to ES or human ESC media. Doxycyline was removed after 15 days and colonies were picked after 3 to 5 more days and maintained as described above.

### Alkaline phosphatase, immunofluorescence assay and FISH

For alkaline phosphatase (AP) staining, cells were fixed with 2% paraformaldehyde for 10 min at RT, washed two times with PBS (Euroclone) and treated with AP solution containing BCIP and NBT (Roche). For immunocytochemistry, cells were fixed with 4% paraformaldehyde for 20 min at RT and washed three times with PBS (Euroclone). Fixed cells were then incubated in blocking buffer containing 5% goat serum (Sigma) and 0,1% Triton X-100 for 30 minutes at RT. The cells were then incubated with primary antibodies diluted in the blocking buffer overnight at 4°C. On the next day, after 3 washes in PBS, the cells were exposed to secondary antibodies at RT for one hour. Primary antibodies included SSEA1 (1∶100, Chemicon), Desmin (1∶200, kindly donated by G.Cossu), SMA (1∶200, kindly donated by L. Naldini), Nanog (1∶100, Abcam), Oct4 (1∶100, Abcam), Sox2 (1∶100, Abcam), me3H3K27 (1∶100, kindly donated by G. Testa). Secondary antibodies used were donkey anti-mouse and anti-rabbit IgG and IgM antibodies conjugated with Alexa 488, Alexa 594 (1∶500, Molecular Probes). Nuclear staining was performed with Hoechst 33342 (Invitrogen). Pictures were taken using a Nikon Eclipse 3000 fluorescence microscope.

For RNA FISH the *Xist* probe used a 19 kb genomic fragment derived from a lamda clone which covers most of *Xist* gene. Probes were labeled by nick translation with Spectrum Green-dUTP and hybridized (0.1 ug of probe DNA with 10 ug of salmon sperm DNA per coverslip) in 50% formamide, 2 x SSC, 20% dextran sulfate, 1 mg/ml BSA (Biolabs), and 200 mM VRC, overnight at 37°C. After three washes in 50% formamide, 2 x SSC and three washes in 2 x SSC at 42°C, DNA was counterstained for 3 min in 0.2 mg/ml DAPI, followed by a final wash in 2 x SSC. Samples were mounted in 90% glycerol, 0.1x PBS, 0.1% p- phenylenediamine.

### Feeder-free culture of FGF-iPSCs

FGF-iPS cells were cultivated on fibronectin (Sigma) coated dishes (5 ug/ml) in a knock-out DMEM medium (Invitrogen) containing N2 and B27 and supplemented with 100 ng/ml bFGF (Invitrogen), 50 ng/ml ActivinA (Peprotech), 2-β-mercaptoethanol, L-glutamine and non-essential amino acids (Invitrogen).

### RT-PCR analysis

The RNeasy mini-kit (Qiagen) was used for total RNA isolation and Superscript reverse transcriptase II (Invitrogen) for cDNA synthesis starting with 2 µg of total RNA. Amplification of specific genes was carried out using primers shown in Table S1. PCR conditions were as follows: 95°C for 10 minutes; denaturation at 95°C for 30 sec, annealing for 30 sec at a temperature specific for each primer set, and extension at 72°C for 30 sec for 33 cycles and then 72°C for 10 minutes.

### Luciferase reporter assay

Plasmids for the Oct4 enhancer assay were cloned as described [2]. 12 well plates with mES, EpiSCs and FGF-iPS cells were transfected with the 1 ug of the respective Oct4 enhancer plasmid and 0.1 ug of a renilla control plasmid using Lipofectamine2000 (Invitrogen). Luciferase activity was measured using the Dual Luciferase Reporter Assay System (Promega) following the manufacturer's protocol. All experiments were carried out in triplicates and normalized to renilla activity to account for differences in transfection efficiencies.

### Agilent chip hybridization

For genome-wide expression analysis, total RNA was extracted using Trizol reagent (Invitrogen) and labeled and hybridized to Agilent Whole Mouse Genome Oligo 4X44K Microarrays (one-color platform) according to the manufacturer's protocols. The gene expression results were analyzed using GeneSifter microarray analysis software.

### Illumina bead chip hybridization

Biotin-labelled cRNA was produced using a linear ampLIFication kit (Ambion, Austin, TX, United States). Total RNA was quality-checked by NanoDrop analysis (NanoDrop Technologies, Wilmington, DE, USA) and a quantity of 400 ng was used as input. Chip hybridization, washing, Cy3-streptavidin staining and scanning were performed on the Illumina BeadStation 500 platform (Illumina, San Diego, CA, United States), according to manufacturer's instruction. cRNA samples were hybridized onto Illumina mouse-8 BeadChips. We hybridized the following samples as biological replicates: ESCs, LIF-iPSCs and FGF-iPSCs. All basic expression data analysis was carried out using the BeadStudio software 3.0. Raw data were background-subtracted and normalized using the “rank invariant” algorithm and then filtered for significant expression on the basis of negative control beads. Pathway analysis was determined according to Gene Ontology terms or mapped to Kegg pathways using DAVID 2006 (http://david.abcc.ncifcrf.gov) by using GenBank accession numbers represented by the corresponding chip oligonucleotides as input. Analysis of the transcriptional regulatory circuit was performed using String database 8.0 (http://string.embl.de/).

### Real-time polymerase chain reaction (qPCR)

Real-Time PCR was performed in 384 Well Optical Reaction Plates (Applied Biosystems, Foster City, CA, United States). The PCR mix in each well included 5 µl of SYBR®Green PCR Master Mix (Applied Biosystems), 1.5 µl each of the forward and reverse primers (5 ng/µl) and 1 µl of single strand cDNA (50 ng/µl) in a final reaction volume of 10 µl. qPCR reactions were carried out on the ABI PRISM 7900HT Sequence Detection System (Applied Biosystems) using the following program: 50°C for 2 min, 95°C for 10 min, 95°C for 15 s and 60°C for 1 min, 95°C for 15 s, 60°C for 15 s and 95°C for 15 s for a total of 40 cycles. Triplicate amplifications were carried out for each target gene with three wells serving as negative controls. The output data generated by the Sequence Detection System software were transferred to Microsoft Excel for analysis. Quantification was performed through a standard curve-based method and normalized to the expression of the housekeeping gene beta-actin. Primer sequences are provided in Table S1.

### Western blotting

Protein extracts were isolated from ESCs, LIF-iPSCs and FGF-iPSCs using a modified RIPA buffer lysis buffer (150 mM NaCl, 10 mM Tris-HCl, 1 mM EDTA, 1% Triton-X100; pH 7.8) supplemented with complete protease inhibitor cocktail (Sigma) added just before use. Protein concentration was determined according to the Bradford method. Equal quantities of proteins were separated by electrophoresis in a 10% SDS-polyacrylamide gel (BioRad, Hercules, CA). Primary antibodies, purchased from Cell Signalling Technology, against SMAD 2/3, Phospho-SMAD 2/3, SMAD 1/5/8, Phospho-SMAD 1/5/8, ERK ½, phospho-ERK ½, Stat3 and phospho-Stat3 were used with the suitable HRP-conjugated secondary antibodies. Bound antibodies on nitrocellulose membrane (Amersham Biosciences) were then detected using the chemiluminescent substrate ECL (Amersham Biosciences).

### Methylation analysis

Methylation analysis was performed as previously described by Blelloch et al. (2006). Genomic DNA extraction was performed using the Blood & Cell Culture DNA Mini Kit (Qiagen). Bisulfite treatment of DNA was achieved using the CpGenome DNA Modification Kit (Chemicon) according to the manufacturer's instructions. The resulting modified DNA was ampLIFied by nested polymerase chain reaction (PCR) using two forward (Met-Oct4 F1 and Met-Oct4 F2) primers and one reverse (Met-Oct4 R) primer listed in Table S1.The first round of PCR was done as follows: 94°C for 4 minutes; five cycles of 94°C for 30 seconds, 56°C for 1 minute (–1°C per cycle), 72°C for 1 minute; and 30 cycles of 94°C for 30 seconds, 51°C for 45 seconds, and 72°C for 1 minute, 20 seconds. The second round of PCR was 94°C for 4 minutes; 30 cycles of 94°C for 30 seconds, 53.5°C for 1 minute, and 72°C for 1 minute 20 seconds. The resulting ampLIFied products were gel-purified (Promega), subcloned into the TA vector (Invitrogen), and sequenced using the T7 and SP6 primers.

### Small-molecule compounds

The following inhibitors were used at the indicated final concentrations: ALK inhibitor (A8301, Tocris Bioscience; 0.5 µM) and JAK inhibitor I (Calbiochem 420099; 0.6 µM), SU5402 (Calbiochem 572630, 10 µM). Anti-mouse LIF antibody was purchased by R&D system.

### Teratoma formation

One million of serially passaged FGF-iPS cells were harvested by collagenase IV treatment, diluted in DMEM/F12 and injected subcutaneously into the hind limb muscle of *SCID* mice. Five to six weeks after the injection all mice developed tumors. Teratomas were removed, fixed overnight in 4% PFA and embedded in Paraffin. 10 um thicken sections were stained with hematoxylin and eosin for conventional morphological assessment.

### Differentiation of FGF-iPS cells

For EB formation, FGF-iPS cells were harvested by treating with 1 mg/ml collagenase IV for one hour. The clumps of cells were then transferred into T25 flasks for 6 days in differentiation medium (knock-out DMEM containing 20% knock-out serum replacement, 2-β-mercaptoethanol, L-glutamine, penicillin-streptomycin and non-essential amino acids). For the generation of cardiomyocytes and Sox17-positive cells, EBs were plated onto gelatin-coated tissue culture dishes at very low density in DMEM medium with 20% animal serum. Medium was replaced every two days. For neural differentiation EBs were cultured for 6 days in differentiation medium and then plated onto matrigel coated dishes in N2-medium.

### Chimera production

Morulas were collected from the oviduct of superovulated CD-1 females mated with CD-1 males 48 hrs earlier by a flushing procedure with BMOC-3 medium. The zona pellucida was removed from the embryos by washing through 3 consecutive drops of Acid Tyrode solution (Sigma). Small clumps of FGF-cells were generated after treatment with collagenase IV. One clump containing between 5 to 15 FGF-iPS cells was added to each embryo and cultured in BMOC-3 medium (Invitrogen) overnight at 37°C 5% in 5% CO(2). The following day, embryos, which had developed into blastocysts, were transferred into the uterus of pseudopregnant recipient CD-1 females mated with vasectomized CD-1 males 2.5 days earlier.

## Supporting Information

Figure S1 **Epigenetic, transgene silencing and chromosome stability of FGF-iPSCs.** (A) Scheme representing the methylation pattern of the Oct4 promoter in FGF-iPSCs (line 5C) and MEFs. Open circles indicate unmethylated, while filled circles depict methylated CpG dinucleotides. All the CpG dinucleotides tested were unmethylated in FGF-iPSCs. (B) Silencing of retroviral expressed transgenes was assessed by qPCR analysis. (C) A chromosome spread of FGF-iPS cells is presented with a normal number of chromosomes (2n = 40). (D) Luciferase reporter assay of Oct4 enhancer usage in murine ES cells, EpiSCs, LIF-derived iPS cells and FGF-derived iPS cells as indicated.
(TIF)

Figure S2 **Reprogramming by lentiviral inducible transduction.** (A) Schematic representation of the strategy used for the reprogramming of murine fibroblasts into iPS cells in the presence of LIF or bFGF. (B) Image of an EpiSC-like iPS cell colony derived in bFGF.
(TIF)

Figure S3 **Effect of FGF withdrawal on FGF-iPSCs.** In the absence of bFGF (B) or in the presence of a specific FGFR inhibitor (SU5402) (C), FGF-iPSCs differentiate extensively.
(TIF)

Figure S4 **Facile conversion of FGF-iPSCs to LIF supplemented culture conditions.** (A–D) After stimulation in a medium containing LIF, FGF-iPSCs assume a close ES-like morphology and adapt to these new conditions growing as homogeneous colonies over time (10 passages, for 3 months in culture). We termed these cells as LIF-stimulated FGF-iPSCs. When these cells were returned back to the original FGF medium and cultured for another week, they acquired the original morphological characteristic (reverted FGF-iPSCs) (E, F). A, C, E, Cell colonies in bright-field. B, D, F, GFP expression controlled by the Oct4 promoter (OG2 transgenic cells).
(TIF)

Figure S5 **Expression analysis on Dlk1-Dio locus.** (A–B) Expression of Glt2 and Dlk1 locus in ES cells, FGF-iPS cells and EpiSC cells. The analysis reveal that in at least one FGF-iPSC clone the expression of Gtl2 is correct.
(TIF)

Movie S1 **Beating cardiomyocytes derived from** ***in vitro*** **differentiation of FGF-iPSCs.**
(MPG)

Table S1 **List of the primers used for RT-PCR, qPCRs and genomic methylation analysis.**
(DOC)
