# Supplementary figures and images for: An ES-Like Pluripotent State in FGF-Dependent Murine iPS cells
Source: PLoS One. 2010 Dec 30;5(12):e16092. doi: 10.1371/journal.pone.0016092 (PMC3012723; doi:10.1371/journal.pone.0016092)

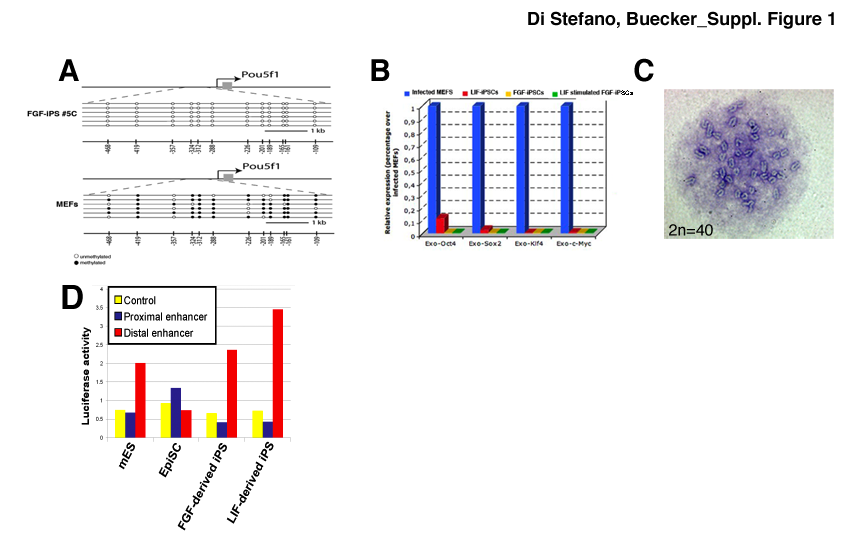

Supplement: Figure S1 — Epigenetic, transgene silencing and chromosome stability of FGF-iPSCs. (A) Scheme representing the methylation pattern of the Oct4 promoter in FGF-iPSCs (line 5C) and MEFs. Open circles indicate unmethylated, while filled circles depict methylated CpG dinucleotides. All the CpG dinucleotides tested were unmethylated in FGF-iPSCs. (B) Silencing of retroviral expressed transgenes was assessed by qPCR analysis. (C) A chromosome spread of FGF-iPS cells is presented with a normal number of chromosomes (2n = 40). (D) Luciferase reporter assay of Oct4 enhancer usage in murine ES cells, EpiSCs, LIF-derived iPS cells and FGF-derived iPS cells as indicated. (TIF) [file pone.0016092.s001.tif]

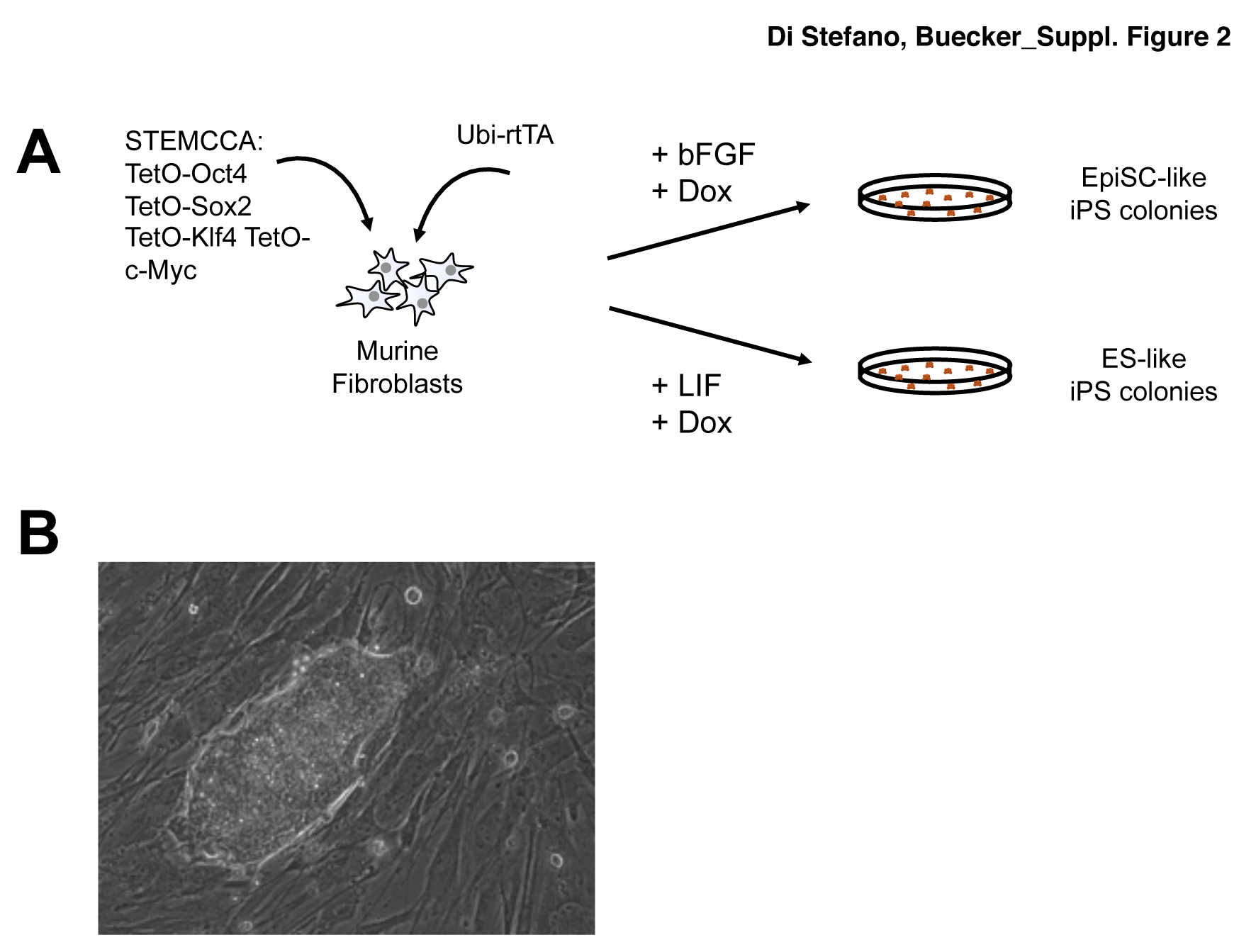

Supplement: Figure S2 — Reprogramming by lentiviral inducible transduction. (A) Schematic representation of the strategy used for the reprogramming of murine fibroblasts into iPS cells in the presence of LIF or bFGF. (B) Image of an EpiSC-like iPS cell colony derived in bFGF. (TIF) [file pone.0016092.s002.tif]

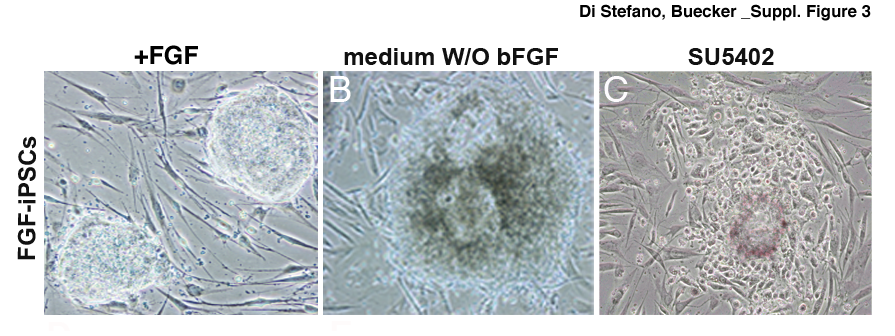

Supplement: Figure S3 — Effect of FGF withdrawal on FGF-iPSCs. In the absence of bFGF (B) or in the presence of a specific FGFR inhibitor (SU5402) (C), FGF-iPSCs differentiate extensively. (TIF) [file pone.0016092.s003.tif]

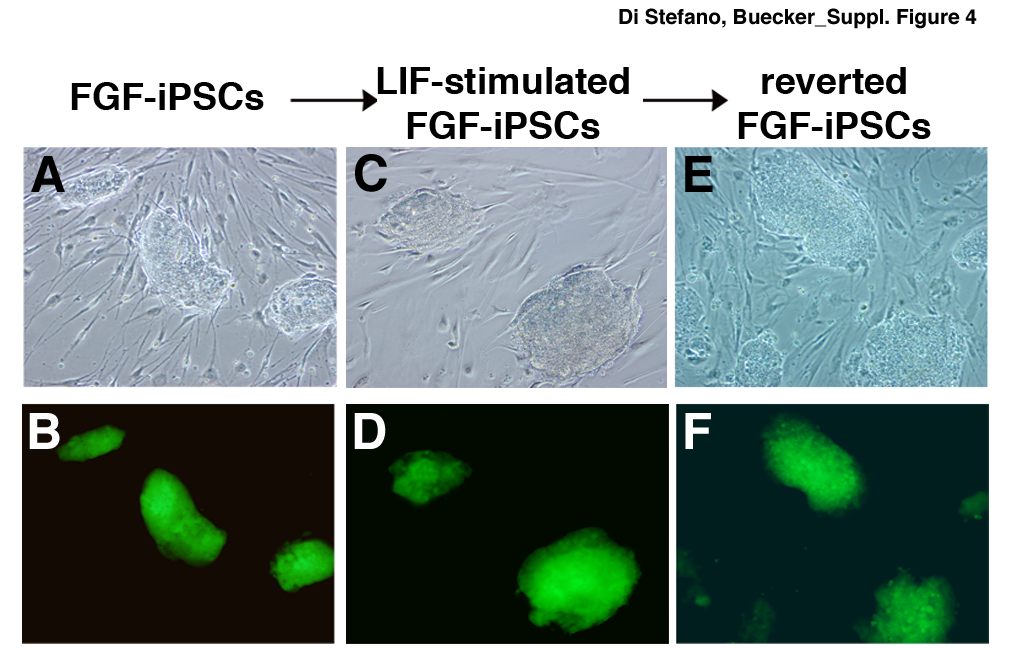

Supplement: Figure S4 — Facile conversion of FGF-iPSCs to LIF supplemented culture conditions. (A–D) After stimulation in a medium containing LIF, FGF-iPSCs assume a close ES-like morphology and adapt to these new conditions growing as homogeneous colonies over time (10 passages, for 3 months in culture). We termed these cells as LIF-stimulated FGF-iPSCs. When these cells were returned back to the original FGF medium and cultured for another week, they acquired the original morphological characteristic (reverted FGF-iPSCs) (E, F). A, C, E, Cell colonies in bright-field. B, D, F, GFP expression controlled by the Oct4 promoter (OG2 transgenic cells). (TIF) [file pone.0016092.s004.tif]

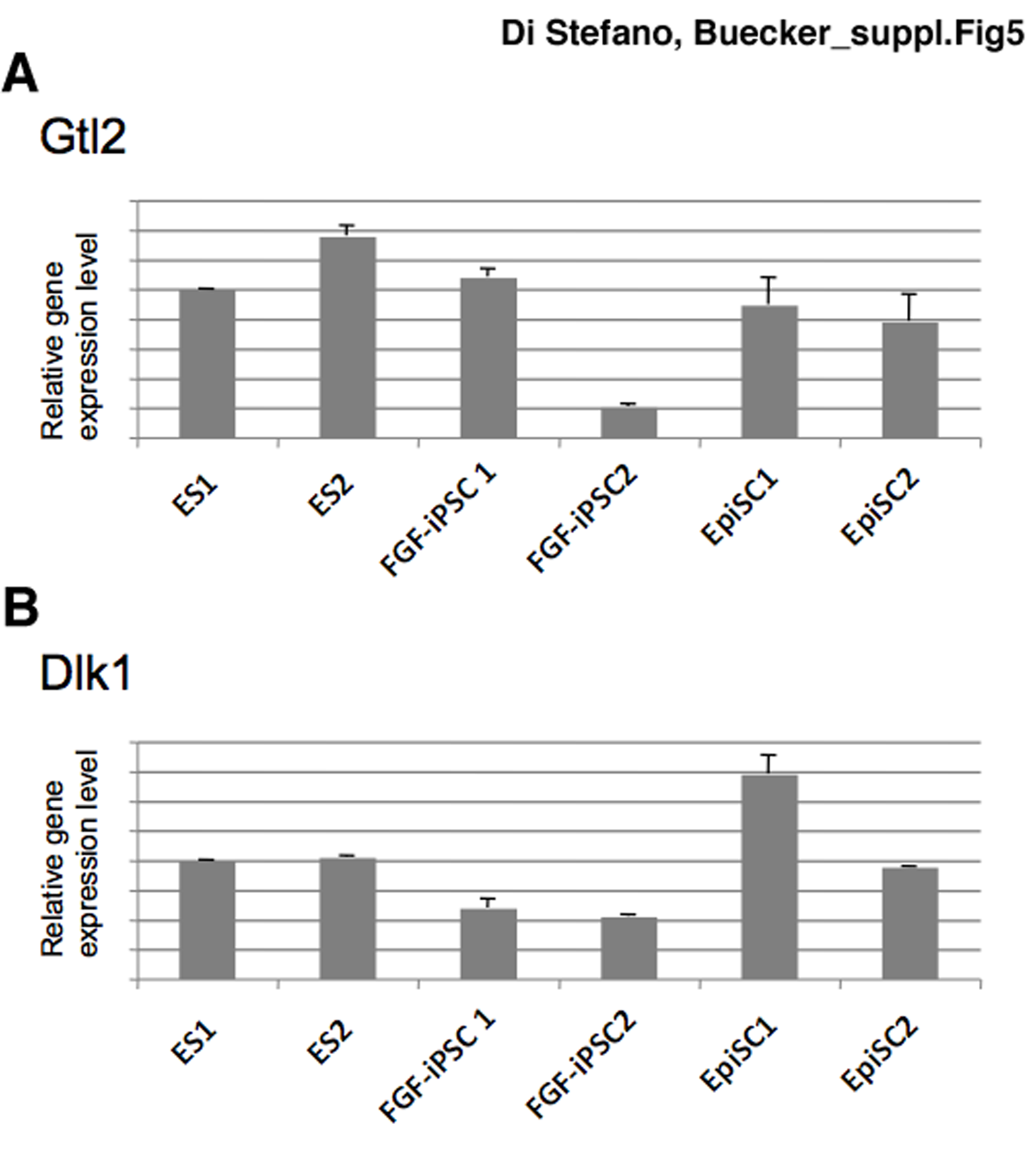

Supplement: Figure S5 — Expression analysis on Dlk1-Dio locus. (A–B) Expression of Glt2 and Dlk1 locus in ES cells, FGF-iPS cells and EpiSC cells. The analysis reveal that in at least one FGF-iPSC clone the expression of Gtl2 is correct. (TIF) [file pone.0016092.s005.tif]
