# Supplementary material for: An ES-Like Pluripotent State in FGF-Dependent Murine iPS cells
Source: PLoS One. 2010 Dec 30;5(12):e16092. doi: 10.1371/journal.pone.0016092 (PMC3012723; doi:10.1371/journal.pone.0016092)
Supplement: Table S1 — List of the primers used for RT-PCR, qPCRs and genomic methylation analysis. (DOC) [file pone.0016092.s007.doc]

| **Genes** | **Forward primer** | **Reverse primer** |
| --- | --- | --- |
|  |  |  |
| **For methylation analysis** | |  |
|  |  |  |
| Met-Oct4 | GTTGTTTTGTTTTGGTTTTGGATAT | CCACCCTCTAACCTTAACCTCTAAC |
|  | ATGGGTTGAAATATTGGGTTTATTTA |  |
|  |  |  |
| **For qPCR** |  |  |
|  |  |  |
| Sall4 | ACACCAATGTGACTCTGCAGG | CGAATCTGTTCCGTAAGCTGG |
|  |  |  |
| Inhab | ATAGAGGACGACATTGGCAGG | GTGCAGTGTCTTCCTGGCTG |
|  |  |  |
| Cer1 | CTCTGGGGAAGGCAGACCTAT | CCACAAACAGATCCGGCTT |
|  |  |  |
| Foxa2 | TAGCGGAGGCAAGAAGACC | CTTAGGCCACCTCGCTTGT |
|  |  |  |
| Gdf3 | ATGCAGCCTTATCAACGGCTT | AGGCGCTTTCTCTAATCCCAG |
|  |  |  |
| Dppa4 | ACGCCAGGACAGACTCGTAG | TGCTGCTCACTCGTTTCTTCT |
|  |  |  |
| Rex1 | TGGAAGCGAGTTCCCTTCTC | GCCGCCTGCAAGTAATGAG |
|  |  |  |
| Endo-Oct4 | CAGCCAGACCACCATCTGTC | GTCTCCGATTTGCATATCTCCTG |
|  |  |  |
| Endo-Sox2 | GCGGAGTGGAAACTTTTGTCC | CGGGAACGCTGTACTTATCCTT |
|  |  |  |
| Grem1 | AAGCGAGATTGGTGCAAAACT | ACCCTTCCTCCTTTCGGATGT |
|  |  |  |
| Nanog | TTGCTTACAAGGGTCTGCTACT | ACTGGTAGAAGAATCAGGGCT |
|  |  |  |
| BMP4 | GACTTCGAGGCGACACTTCTA | GCCGGTAAAGATCCCTCATGTAA |
|  |  |  |
| Nodal | TTCAAGCCTGTTGGGCTCTAC | TCCGGTCACGTCCACATCTT |
|  |  |  |
| Otx2 | TATCTAAAGCAACCGCCTTACG | AAGTCCATACCCGAAGTGGTC |
|  |  |  |
| Lefty | CCAACCGCACTGCCCTTAT | CGCGAAACGAACCAACTTGT |
|  |  |  |
| FGF5 | TGTGTCTCAGGGGATTGTAGG | AGCTGTTTTCTTGGAATCTCTCC |
|  |  |  |
| Beta-Actin | GGCTGTATTCCCCTCCATGC | CCAGTTGGTAACAATGCCATGT |
|  |  |  |
| Cripto | GATAACAGCATTTGGGACCAGA | GGTCGTCACAGACGGCGTTTGAC |
|  |  |  |
| Eras | CCTCATCAGACTGCTACTCCTG | CCTGCCCAGATGTATCCAGAAC |
|  |  |  |
| Jak1 | CTCTCTGTCACAACCTCTTCGC | TTGGTAAAGTAGAACCTCATGCG |
|  |  |  |
| Stat3 | TGGCACCTTGGATTGAGAGTC | GCAGGAATCGGCTATATTGCT |
|  |  |  |
| Pim1 | CTGGCCCGAGGATTCTTCTG | CGGCTCAGGTCGATTAAGATGTT |
|  |  |  |
| Sox17 | GATGCGGGATACGCCAGTG | CCACCACCTCGCCTTTCAC |
|  |  |  |
| Gata6 | GGCAGTGTGAGTGGAGGTG | TGGTACGTTCCGTTCAGCG |
|  |  |  |
| ex-Oct4 | TACACCCTAAGCCTCCGCCT | GGTGGAAAGACGGCTCAAAG |
|  |  |  |
| ex-Sox2 | ATCCAGCCCTCACTCCTTCTC | GGTGGAAAGACGGCTCAAAG |
|  |  |  |
| ex-Klf4 | TCTCTAGGCGCCGGAATTC | CCATGTCAGACTCGCCAGGT |
|  |  |  |
| ex-c-Myc | CTTCTCTAGGCGCCGGAATT | TGGTGAAGTTCACGTTGAGGG |
|  |  |  |
| Beta-Actin | GGCATCGTGATGGACTCCG | GCTGGAAGGTGGACAGCGA |
